# One-pot, light-induced, liquid crystal-templated synthesis of nanoporous silver films at room temperature

**DOI:** 10.55730/1300-0527.3761

**Published:** 2025-06-04

**Authors:** Fadime MERT BALCI

**Affiliations:** Department of Chemistry, Izmir Institute of Technology, İzmir, Turkiye

**Keywords:** Porous silver film, silver nanoparticles, lyotropic liquid crystal, surface plasmon polaritons, one-pot synthesis, photochemistry

## Abstract

Nanoporous silver (NPS) films, characterized by a 3-dimensional bicontinuous structure of interconnected nanopores and ligaments, have found widespread use in spectroscopy, plasmonics, solar cells, catalysis, and chemical sensing. Traditionally, NPS films are fabricated via chemical dealloying, where a less noble metal (e.g., Cu or Al) is selectively removed through harsh chemical etching. However, residual traces of these metals can adversely affect the performance of NPS thin films in applications such as plasmonics and catalysis. This paper reports a one-pot, liquid crystal-templated method for synthesizing ultrapure NPS thin films at room temperature for the first time. The process begins with the preparation of an LLC composed of a nonionic surfactant and AgNO_3_ that is then coated onto solid substrates. Exposure of the LLC film to ultraviolet light facilitates the in situ synthesis of Ag nanoparticles within the liquid crystal film. Subsequent solvent washing removes the surfactant molecules and any unreacted metal ions, yielding NPS films comprised of densely packed Ag nanoparticles on glass substrates. The resulting NPS films feature a 3-dimensional structure with uniformly distributed, interconnected nanopores. Synthesized under ambient conditions and scalable over large areas, these ultrapure NPS films present a highly promising platform for advanced applications in catalysis, spectroscopy, plasmonics, and biosensing.

## 1. Introduction

Nanoporous materials have either periodic or stochastic pore structures and may be composed of organic, inorganic, or hybrid constituents [1]. These materials typically have pore sizes ranging from 2 nm to over 50 nm, encompassing pore sizes ranging from the microporous to the macroporous scale, and have an exceptionally high surface area relative to their bulk volume. Owing to these unique structural characteristics, nanoporous materials with specific pore sizes have exceptional functionality in diverse technological domains, including catalysis, spectroscopy, sensors, filtration, solar cells, and metal-ion batteries [2]. For example, zeolites, which are crystalline nanoporous aluminosilicates composed of TO_4_ tetrahedra (where T = Al or Si), possess an ordered arrangement of nanopores with molecular dimensions that make them among the most important catalysts used in the chemical and petrochemical industries [3]. Among nanoporous materials, nanoporous noble metal thin films such as gold (Au) [4], silver (Ag) [5], and platinum (Pt) [6] are very attractive due to their fascinating physical (e.g., optical) and chemical (e.g., catalytic) properties. Nanoporous noble metal thin films contain a high density of nanoscale pores and ligaments that are randomly arranged within 3-dimensional frameworks [7]. Importantly, silver and gold nanoparticles, along with nanovoids within nanoporous thin films, support surface plasmon polaritons (SPPs), whose resonance wavelengths can be altered by adjusting the composition, size, and shape of the nanoparticles, as well as the surrounding dielectric environment [8]. The ligaments and nanovoids within the nanoporous noble metal thin films act as plasmonic nanocavities, enabling the strong confinement of incident light at the nanoscale and significantly enhancing its interaction with matter placed within the nanocavities. Notably, nanoporous noble metal thin films feature densely distributed regions of enhanced electromagnetic fields, known as plasmonic hot spots, which originate from collective plasmonic interactions and geometric confinement within their complex nanostructured frameworks [9]. For example, silver or gold nanostructures greatly enhance the vibrational modes of molecules placed near them [10]. Indeed, surface-enhanced Raman scattering (SERS) allows for the spectroscopic identification of single molecules localized within individual plasmonic cavities, highlighting its remarkable sensitivity at the nanoscale [10,11]. Nanoporous Ag is an ideal SERS substrate compared to Au or Pt due to its exceptionally strong Raman signal enhancement and the relatively low cost of Ag. Transforming bulk silver into a nanoporous silver (NPS) film leads to enhanced and often unexpected chemical (e.g., catalytic) and physical (e.g., optical) properties. Consequently, there has been a growing interest in the fabrication of NPS thin films through various techniques, including chemical dealloying [12], plasma treatment [13], electrochemical deposition [14], sputtering [15], and hard or soft templating [16]. For example, nanoporous Cu-Ag alloy films have been electrodeposited onto carbon paper substrates from plating bath solutions containing CuSO_4_ and Ag_2_SO_4_ as metal sources, along with 3,5-diamino-1,2,4-triazole as an inhibitor, highlighting their suitability for electrochemical CO_2_ conversion applications [14]. Another widely used technique for the fabrication of NPS films is chemical dealloying, in which the less noble element, such as Cu or Al, is selectively removed from the alloy film. As an example, Qiu et al. [12] reported the synthesis of robust monolithic NPS structures by chemically dealloying Ag-Al alloys. Crucially, they found that HCl solution, rather than NaOH solution, serves as a more effective dealloying agent for the fabrication of NPS films. Additionally, the structural features of NPS films, such as ligament and pore dimensions, can be controlled by modifying the acid concentration or altering the duration of the dealloying process [12]. In another study, an NPS film serving as a SERS substrate was fabricated via successive O_2_ and Ar plasma treatments on a planar silver film-polystyrene substrate [13]. The main advantage of this approach is that NPS films can be formed without the use of harsh etching chemicals or the high temperatures typically required in chemical dealloying techniques [13]. Furthermore, in the fabrication of NPS films, densely packed NaCl particles served as a hard template into which an Al-Ag alloy was introduced at elevated temperatures, with the Al-rich regions functioning as a soft template. Template removal was accomplished by dissolving NaCl in water and by chemically dealloying the Al component using either a strong acid (HCl) or a strong base (NaOH) [16]. However, the widely used dealloying technique for producing NPS films typically requires high temperature annealing to form alloy films (e.g., Al-Ag or Cu-Ag alloy films), along with harsh etching involving corrosive and strong acidic or basic chemicals. In addition, NPS films fabricated via chemical dealloying techniques often contain metallic impurities, such as Al or Cu, resulting in bimetallic structures that limit their effectiveness in plasmonic or catalytic applications. For example, the inclusion of Al impurities in the NPS films led to a marked reduction in the Raman signal intensity at 1366 cm^−1^ corresponding to rhodamine 6G molecules [12]. Therefore, the residual nonnoble metal impurities in NPS films, resulting from a chemical dealloying process, reduce the performance of NPS films in plasmonic applications. An alternative approach for fabricating nanoporous noble metal thin films is liquid crystal templating, as shown previously [17]. In this approach, an aqueous HAuCl_4_ solution was incorporated into a hexagonal lyotropic liquid crystal (LLC) system consisting of a strong acid and a nonionic surfactant, enabling the in situ photochemical formation of gold nanoparticles within the liquid crystal matrix [17]. Subsequent high-temperature calcination of the Au NP-loaded films on solid substrates yielded large-area, high-purity nanoporous gold (NPG) films.

**Figure 1:**
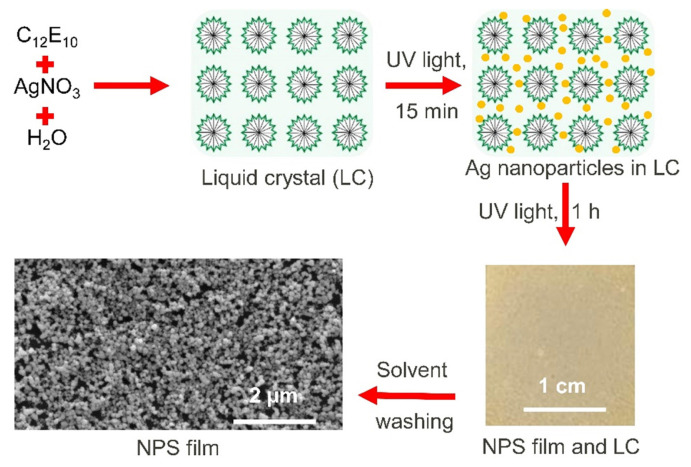
Room-temperature fabrication of NPS thin films via light-driven synthesis guided by a liquid crystal template. After spin coating the LLC solution containing 10-lauryl ether and AgNO_3_ onto solid substrates, a thin LLC film is formed. The LLC film is exposed to UV light to synthesize Ag nanoparticles within the LLC film. The color of the LLC film during the fabrication process is initially colorless and translucent. It then changes to yellow as Ag nanoparticles form and ultimately becomes a gray-yellow hue, indicating the formation of the NPS film. Subsequent solvent washing removes the surfactant molecules and any unreacted silver ions remaining in the LLC film. The SEM images showed that the fabricated NPS films are highly nanoporous.

Herein, this paper reports a one-pot, light-driven, liquid crystal-templated approach for the synthesis of ultrapure NPS thin films at room temperature for the first time. As illustrated in Figure 1, the synthesis of NPS films begins with the preparation of a hexagonal LLC mesophase composed of AgNO_3_ and a nonionic surfactant. Polarized optical microscopy (POM) (showing texture formation) and small-angle X-ray diffraction (XRD) (showing diffractions at small angles) confirm the formation of the LLC mesophase of the surfactant and silver salt. Ag nanoparticles within the LLC film are photochemically synthesized by exposing the LLC film, which contains silver salt, to ultraviolet (UV) light. The growth of Ag nanoparticles, supporting a localized surface plasmon resonance wavelength at around 425 nm, is monitored using UV-visible absorption spectroscopy. Numerous Ag nanoparticles are formed without disrupting the liquid crystal structure after UV light exposure, with optical extinction measurements indicating Ag NP formation within approximately 5 min of exposure. POM observations indicate that the LLC film containing Ag nanoparticles retains its liquid crystalline phase after UV light exposure, at least for the duration of the experiment. Importantly, the NPS films are obtained at room temperature by solvent washing of the LLC film, which contains a high-density Ag nanoparticle. The resulting ultrapure (monometallic) NPS films are highly nanoporous, with Ag nanoparticles and nanovoids (less than 100 nm in size) arranged in a 3-dimensional structure (Figure 1). Therefore, these NPS thin films can be used as ultrasensitive SERS substrates for detecting extremely low concentrations of analyte molecules when placed near the plasmonic cavities within the NPS film. For example, in previous studies, NPS films fabricated using the dealloying process were used as SERS substrates for detecting extremely low concentrations of analyte molecules [12].

## 2. Materials and methods

### 2.1. Chemicals

All reagents were procured from commercial sources and utilized without additional purification. Decaethylene glycol monododecyl ether (10-lauryl ether, C_12_E_10_) and silver nitrate (AgNO_3_) were sourced from Sigma-Aldrich (St Louis, MO, USA), while isopropyl alcohol (IPA, 99.5% purity) was supplied by Tekkim (Istanbul, Türkiye). All experimental procedures used Milli-Q water with a resistivity of 18.2 MΩ·cm at 25 °C. Glass substrates were cleaned with a piranha solution (a 3:1 mixture of H_2_SO_4_ and H_2_O_2_ by volume), followed by multiple rinses with Milli-Q water and subsequent drying.

### 2.2. Fabrication of NPS films

In a typical preparation of the hexagonal LLC phase, 0.5 g of nonionic surfactant (10-lauryl ether, C_12_E_10_) was dissolved in 0.5 mL of isopropyl alcohol, followed by the addition of 1 mL of 2 M AgNO_3_ aqueous solution. After the addition of each component, the mixture was vortexed for approximately 1 min. The resulting LLC solution was then spin coated onto glass substrates at various rotation speeds. Once the LLC phase was formed, the gel-like samples on glass substrates were exposed to UV light (THORLABS, M365L2-C1, Newton, NJ, USA) for varying durations. The UV light source have a total optical power of 120 mW, and a maximum emission at around 365 nm. Spherical silver nanoparticles, interconnected within the LLC medium, were formed upon UV irradiation. After the photochemical reaction, the LLC films containing Ag nanoparticles were washed 3 times with IPA (solvent washing) to remove surfactants, yielding surfactant-free NPS films.

### 2.3. Characterization of NPS films

All characterization procedures were carried out under ambient environmental conditions. To track the growth of silver nanoparticles within the LLC phase, optical extinction measurements were performed directly on glass substrates, without extracting the nanoparticles from the LLC matrix. The glass substrates were spin coated with the LLC mixture, exposed to UV light for controlled time intervals, and then analyzed at specific reaction times using extinction spectroscopy. For these optical measurements, a deuterium-tungsten halogen light source (DH2000-BAL, Ocean Optics, Orlando, FL, USA) and a spectrometer (USB4000, Ocean Optics) were utilized. Following synthesis of silver nanoparticles, the NPS films were separated from the liquid crystal medium through solvent rinsing for further structural and morphological investigation. Surface morphology was studied using a scanning electron microscopy (SEM, Quanta 250, FEI, Hillsboro, OR, USA), while crystallographic information was obtained via X-ray diffraction (XRD) using a Philips X’Pert Pro diffractometer (Amsterdam, Netherlands) equipped with a Cu-Kα radiation source (λ = 1.54056 Å).

## 3. Results and discussion

**Figure 2:**
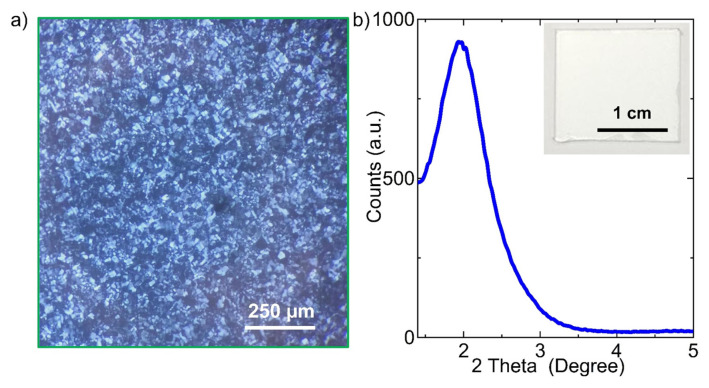
LLC mesophase formation using silver nitrate salt and surfactant molecules. (a) POM image of an LLC film formed after spin coating the LLC solution onto a glass substrate. A characteristic optical fan texture of the liquid crystal phase is observed under crossed polarizers. (b) Low-angle XRD pattern of the liquid crystal film. The inset displays an image of a large-scale LLC film deposited on a glass substrate.

Figure 1 illustrates the room-temperature fabrication process of NPS thin films through light-driven synthesis directed by a liquid crystal template. To photochemically synthesize Ag nanoparticles within the LLC mesophase, an LLC mesophase consisting of 10-lauryl ether (C_12_E_10_) and AgNO_3_ (with a 2.5 mole ratio of AgNO_3_/C_12_E_10_) in a water-isopropanol mixture was prepared. The freshly prepared uniform LLC solution was coated onto solid substrates, and as the isopropanol solvent evaporated, a well-ordered hexagonal mesophase formed uniformly across the glass surface, ensuring complete coverage. This is confirmed by the fan-like texture formation observed under POM, as shown in Figure 2a. It should be noted that isotropic samples (e.g., water) have uniform optical properties in all directions, whereas anisotropic samples (e.g., hexagonal LLCs) do not. As a result, the hexagonal LLC mesophase of C_12_E_10_/AgNO_3_ used in this study displays fan-like texture when observed between crossed polarizers. Additionally, the formation of LLC mesophase was verified through low-angle XRD measurements conducted on thin films coated onto glass substrates, as presented in Figure 2b. The LLC mesophase film gives a low-angle diffraction peak at around 2°, 2θ. Both XRD and POM observations verify that the LLC mesophase of C_12_E_10_/AgNO_3_ has a hexagonal structure. The inset in Figure 2b shows a photograph of the LLC mesophase before exposure to the UV light. POM observations confirm that the LLC mesophase remains highly stable both during and after UV light exposure. Indeed, the LLC mesophase retains its stability for several days under ambient conditions. Presumably, similar to the sulfuric acid/C_12_E_10_ hexagonal LLC mesophase system, which has been extensively studied previously, the C_12_E_10_/AgNO_3_ hexagonal LLC mesophase likely undergoes reversible water molecule exchange with the environment. This significantly contributes to its high stability under ambient conditions [18]. As a result, the liquid crystal mesophase of C_12_E_10_/AgNO_3_ remains stable under ambient conditions for several days during the study period. In a previous study, a lyotropic AgNO_3_-silica liquid crystalline phase was prepared and used as a supramolecular template for the synthesis of Ag nanoparticles within the mesoporous channels and void spaces of the silica [19].

**Figure 3:**
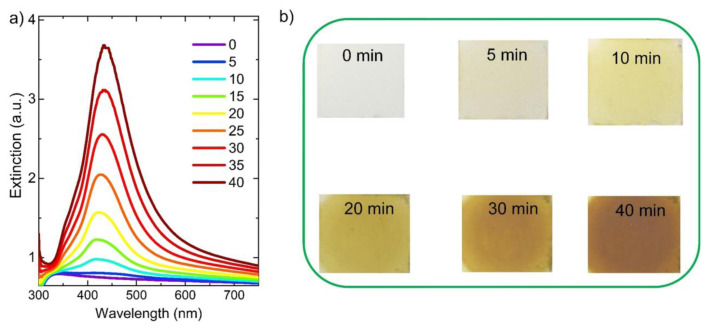
Photochemical growth of Ag nanoparticles within the liquid crystal matrix. (a) Extinction spectra of the LLC film recorded after UV illumination for time intervals between 0 and 40 min. A distinct localized plasmon resonance peak associated with Ag nanoparticles emerged near 425 nm. The appearance of this peak after only 5 min of exposure indicates nanoparticle formation, with a progressive red shift in the peak position observed with extended UV irradiation. (b) Photographic images showing the visual change in the film before and after UV treatment. The initially translucent film adopts a yellow hue upon irradiation, which intensifies to dark yellow as exposure time increases.

Silver nanoparticles were generated within the LLC matrix by subjecting the LLC thin film-coated glass substrate to UV irradiation. It is worth noting that the uniform LLC mesophase film of C_12_E_10_/AgNO_3_ on the glass substrate appears as a blurry white layer that strongly scatters incident light, as illustrated in the inset of Figure 2b. Figure 3a shows the extinction spectra of the liquid crystal film following UV light exposure for durations ranging from 0 to 40 min. It is important to note that spherical (isotropic) Ag nanoparticles have a localized surface plasmon polariton (LSPP) resonance in the blue region of the visible spectrum, around 400 nm [20]. The LSPP resonance wavelength of the Ag nanoparticles can be altered by changing the size, shape, composition, and dielectric environment of the Ag nanoparticles. As an example, the resonant wavelength associated with the localized plasmon modes in spherical isotropic silver nanoparticles can be tuned by transforming them into anisotropic nanoprism structures via a seed-mediated colloidal synthesis performed at room temperature [20]. As shown in Figure 3a, the LSPP resonance wavelength of Ag nanoparticles initially appears around 420 nm. After 10 min of UV light exposure, it shifts to approximately 435 nm and continues to red shift with prolonged exposure up to 40 min. Notably, the formation of Ag NP becomes discernible after just 5 min of UV exposure of the LLC film. Earlier research has clearly shown that nonionic surfactants are capable of reducing silver ions (Ag^+^) to metallic silver (Ag^0^) through oxidation of oxyethylene groups within their molecular backbone to hydroperoxides, even without exposure to visible light [21]. However, this process occurs at a very slow rate, with silver nanoparticle formation taking several hours after mixing the nonionic surfactant with silver ions. In this study, a nonionic surfactant, 10-lauryl ether, was used to prepare the LLC phase containing AgNO_3_. Under these conditions, the surfactant molecules facilitate the reduction of Ag^+^ to Ag^0^. Furthermore, upon exposure to UV light, the reaction rate is significantly enhanced due to the generation of high energy electrons, known as hot electrons, following the plasmonic excitation of silver nanoparticles [22]. It is noteworthy that both silver and gold nanoparticles can produce plasmonic hot electrons when excited by UV or visible light [23]. It is evident that the LSPP resonance wavelength progressively shifts to longer wavelengths with increasing exposure time. A similar trend in nanoparticle formation upon UV light exposure was observed previously with Au nanoparticles [17]. This change was attributed to the increase in size and aggregation of Au nanoparticles resulting from prolonged UV light exposure. It is important to emphasize that the LSPP resonance wavelength red shifts with increasing nanoparticle size and aggregation along the direction of electric field polarization, whereas under perpendicular polarization, it has a blue shift [17]. Figure 3a clearly shows that the intensity of the LSPP resonance wavelength of Ag nanoparticles increases with longer UV light exposure, indicating a growing number of Ag nanoparticles. This trend is further supported by the visual changes in the LLC film shown in Figure 3b; the film initially appears blurry prior to UV light exposure, then gradually turns yellow, and eventually deepens to a dark yellow hue. Previous research has shown that visible light can induce the transformation of spherical Ag nanoparticles into Ag nanoplates, causing a shift of the LSPP resonance wavelength into the visible spectrum [24]. In our recent work, a hexagonal LLC containing gold ions was utilized to synthesize gold microplates via visible light irradiation [25]. Another recent study involved exposing a liquid crystal film embedded with gold ions to UV light, followed by high-temperature calcination to fabricate large-area nanoporous gold thin films [17]. The present study reports, for the first time, the room-temperature, light-driven, liquid crystal-templated synthesis of NPS films.

**Figure 4:**
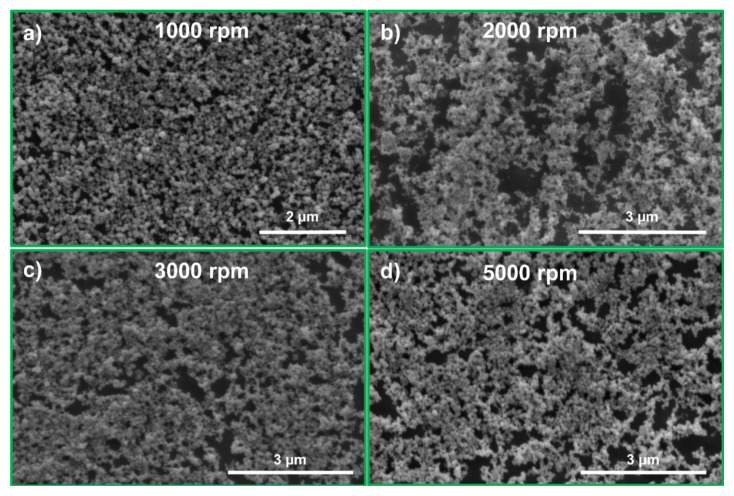
SEM images illustrate the NPS films obtained from the LLC films spin coated on glass substrates at various spinning speeds: (a) 1000 rpm, (b) 2000 rpm, (c) 3000 rpm, and (d) 5000 rpm. As the coating speed increases, the thickness of the liquid crystal film deposited on the substrate is expected to decrease. Uniform NPS film formation is observed in the thicker NPS films produced at 1000 rpm as shown in (a).

**Figure 5:**
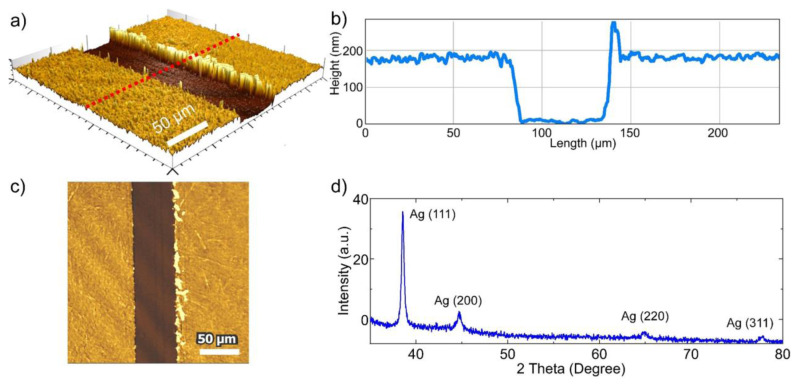
Thickness and microstructural characterization of the NPS film. (a) 3D optical profilometer image of the NPS film. (b) Line scan along the red line in (a), showing an average film thickness of approximately 170 nm for the sample fabricated by spin coating at 1000 rpm. (c) 2D optical profilometer image of the NPS film. (d) XRD pattern of the NPS film, confirming a face-centered cubic crystal structure of silver.

To investigate the effect of the LLC mesophase film thickness on the quality of the NPS film, the LLC mesophase thickness was varied by spin coating the LLC solution at different speeds. More uniform NPS films were obtained from the LLC mesophase films fabricated by spin coating at 1000 rpm, as shown in Figure 4. The thickness of the fabricated NPS film was determined through measurements taken with an optical profilometer. As shown in Figures 5a–5c, the line scan indicates an average thickness of approximately 170 nm for the NPS film fabricated by spin coating the LLC solution at 1000 rpm, with the film also having excellent microscale uniformity.

**Figure 6:**
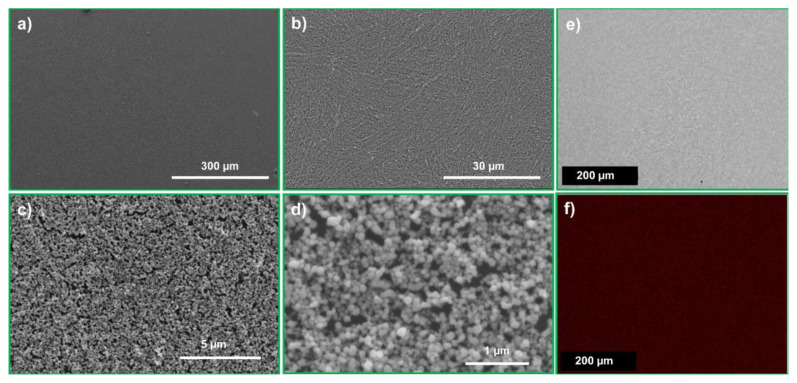
Microstructure and elemental mapping of the NPS films. (a) Low-magnification SEM image showing the uniformity of the NPS film over a large area. (b)–(d) Sequential higher-magnification SEM images of the area shown in (a), showing increasing structural detail. (d) At the highest magnification, numerous aggregated Ag nanoparticles are visible. (e) SEM image of the NPS film corresponding to the area analyzed in (f). (f) Elemental mapping of Ag using the Ag-L signal (in red), showing the high purity and uniformity of silver across the mapped region.

The microstructure of the NPS films was characterized using XRD, SEM, and energy dispersive X-ray spectroscopy (EDX). The XRD pattern shown in Figure 5d has the characteristic peaks of metallic Ag, with a preferred orientation corresponding to the (111) diffraction peak at approximately 2θ = 38.6° [13]. Additionally, diffraction peaks corresponding to other crystallographic planes were observed at approximately 2θ = 44.7° (200), 64.9° (220), and 77.8° (311) [13]. SEM analysis shows that the NPS film features a 3-dimensional nanostructured framework, consisting of nanoscale pores uniformly distributed across the entire silver film, as illustrated in the detailed SEM images in Figure 6. The NPS film contains a high density of uniformly distributed Ag nanoparticles and nanoholes, each with sizes below approximately 100 nm. Figure 6f shows the Ag L signal corresponding to the NPS film in Figure 6e. The EDX mapping in Figure 6f confirms that silver is uniformly distributed across the film surface. These findings indicate that the NPS film is composed of ultrapure (100%) Ag with a highly uniform surface distribution. In previous studies, the commonly used dealloying technique left impurity atoms in the nanoporous metal films that significantly reduced their performance in certain applications [26]. For example, for plasmonics applications, particularly as a SERS substrate, a pure NPS film is highly desirable, as the impurity atoms can significantly decrease its SERS performance [12]. In this study, only Ag was used as the metal source. In contrast, most previous studies have fabricated NPS films via a dealloying process, in which Al or Cu atoms are etched out. However, this process leaves behind residual Al or Cu impurities in the resulting NPS films.

In conclusion, this paper presents a novel one-pot, light-driven, liquid crystal-templated technique for synthesizing ultrapure NPS thin films at room temperature. This approach has several key advantages over previous approaches: 1) the formation of a stable hexagonal LLC phase composed of a nonionic surfactant and AgNO_3_, 2) one-pot synthesis of NPS films at ambient conditions, 3) light-driven synthesis of silver nanoparticles within the liquid crystal matrix, and 4) the synthesis of large-area NPS thin films. Importantly, silver nanoparticles are synthesized in situ within the liquid crystal matrix, and subsequent solvent washing yields ultrapure NPS films. These films have a 3-dimensional network of uniformly distributed, interconnected nanopores. The ultrapure NPS films hold great potential for applications in Raman spectroscopy, nanophotonics, biosensing, and photovoltaics. For instance, the abundant plasmonic cavities within the NPS structure markedly enhance SERS signals of probe molecules such as rhodamine 6G. Furthermore, the nanoscale structure of the NPS film enables the exploration of light-matter interactions, such as plasmon-exciton coupling [20] within the plasmonic cavities. Thus, the high-purity, structurally well-defined NPS films developed in this study have the potential to enable these and other emerging applications.
